# Molecular dynamics simulations of atmospherically relevant molecular clusters: a case study of nitrate ion complexes†

**DOI:** 10.1039/d5cp00908a

**Published:** 2025-05-01

**Authors:** Christopher David Daub, Theo Kurtén, Matti Rissanen

**Affiliations:** a Aerosol Physics Laboratory, Physics Unit, Faculty of Engineering and Natural Sciences, Tampere University 33720 Tampere Finland christopher.daub@tuni.fi; b Department of Chemistry, University of Helsinki P.O. Box 55 Helsinki 00014 Finland

## Abstract

The formation and decomposition of complexes of ions with atmospheric analyte molecules are key processes in chemical ionization mass spectrometry (CIMS) instruments, as well as in atmospheric new particle formation (NPF). In this study, we conduct extensive molecular dynamics (MD) simulations of the decomposition of already-formed molecular complexes with nitrate ions (NO_3_^−^). We study purely thermal decomposition *in vacuo* and in the presence of nitrogen gas, as well as the decomposition driven by electric-field induced collisions with nitrogen gas. Our findings are relevant to improving the understanding of basic processes taking place in CIMS as well as in other MS instruments more generally.

## Abstract 2

Simulated lifetimes of catechol–NO_3_^−^ clusters *in vacuo* and in electric field + bath gas.

## Graphical abstract

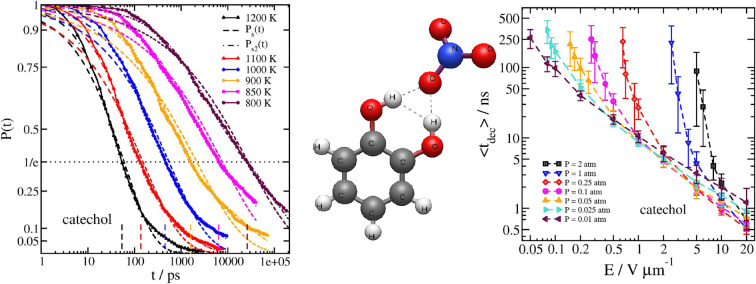

## 1 Introduction

Chemical ionization mass spectrometry (CIMS) instruments are a key component of the toolbox employed to detect trace gases in the atmosphere^1^ or in related laboratory settings (*e.g.* flow reactor setups).^2^ By flowing a reagent gas through an ion source, reagent ions are produced which, after being guided into the ion–molecule reaction region (IMR) with an electric field, can then form clusters with the gaseous sample of interest. If these clusters have sufficient binding energy, they remain stable in the ionization region of the instrument until they can be driven into a mass spectrometer for analysis.^3,4^

Development efforts to improve the sensitivity of CIMS instruments, for example by maintaining the stability of clusters with lower binding energy, continue apace.^5–7^ However, theoretical or computational efforts to model the key processes have lagged behind. Quantum chemistry to predict the binding energy of different clusters has been done,^8–12^ and is a key tool used to help analyze and interpret experimental results.^13,14^ At the same time, focusing only on the binding energy neglects the key contribution of dynamical and transport processes in the experimental setups, as well as for understanding the role of complexation in atmospheric new particle formation (NPF). Recent modelling studies using statistical collision theory^15–17^ and computational fluid dynamics methods^18^ have been insightful, but these lack molecular-level insights. Molecular simulations, meanwhile, have been used to study the mobility of ions in gases,^19,20^ and to model the related phenomenon of solvent loss in electrospray ionization mass spectrometry (ESI-MS).^21^

In this study, we use simple force-field based molecular dynamics (MD) simulations to model the decomposition of ion–molecule complexes. We study two main decomposition mechanisms. First, we simulate the thermal decomposition of complexes prepared with a given initial temperature, both *in vacuo* as well as in the presence of nitrogen bath gas. Second, we come closer to simulating an actual experiment by accelerating the charged complexes in a constant (DC) electric field, and allowing collisions with nitrogen gas to induce the decomposition.

We only study clusters with a nitrate ion, since this is the most important reagent ion currently used in atmospherically relevant CIMS studies.^7,13,14,22,23^ Nitrate ion clusters are very strongly bound, so assuming their initial formation in our simulations is justified. A wide range of different molecules have been detected in NO_3_^−^ CIMS experiments,^13,24^ both in the field and in laboratory settings. In this study, we focus on simpler molecules, as opposed to highly functionalized and poorly characterized highly oxygenated molecules (HOMs) typically targeted in field measurements. Further laboratory experiments to quantify the behaviour of these simpler nitrate complexes will be aided by this computational work.

We chose as analyte molecules the three isomers of dihydroxybenzene (catechol, resorcinol, hydroquinone), 4-nitrophenol, and nitric acid. Although the binding energies of these clusters have been studied by quantum chemistry,^8,10^ to the best of our knowledge this is the first time the physics of the cluster decomposition has been studied by computational methods at the molecular level.

## 2 Methods and models

For the nitric acid molecule, we based our force field on one developed and used previously.^25,26^ However, this force field lacked an O–N–O–H dihedral potential to control the orientation of the OH group. We did a scan of the O–N–O–H dihedral angle using density functional theory (DFT) calculations to determine a reasonable dihedral angle potential term. We also increased the partial charges on the OH group by ±0.05*e* to increase the binding energy with the nitrate ion. For the other analyte molecules, in all cases we used the LigParGen webserver^27^ to generate an optimized potentials for liquid simulations (OPLS)-based force field with partial charges computed at the localized bond-corrected charges (LBCC) level.^28–30^ Nitrogen gas was modeled as a simple nonpolar diatomic molecule with a rigid bond, as developed and used previously.^31,32^ Finally, we used a previously developed model for the nitrate ion.^33^ However, we found that this model combined with the OPLS models for the analytes considerably underestimated the intermolecular binding energy. This was rectified by increasing the partial charges on the atoms in the nitrate ion in all simulations to *q*_N_ = 1.25*e* and *q*_O_ = −0.75*e*.

**Fig. 1:**
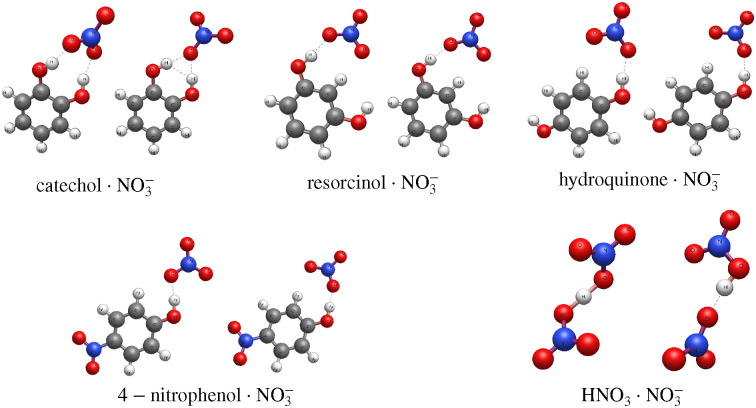
Optimized geometries for nitrate complexes obtained by DFT with the ωB97X-D4 functional and the aug-cc-pVTZ basis set (left side of each pair), and by MD simulation at 5 K with the empirical force field described in the text (right).

The optimized complex geometries (Fig. 1) and binding energies (Table 1) for our empirical force field agree reasonably well with those obtained by quantum chemical methods using ORCA version 6.0.^34,35^ We note that the nitric acid–nitrate ion cluster features a bridging hydrogen which cannot be correctly modelled by a simple empirical force field, although the binding energy agrees well. Importantly, the same trend for binding energy between different dihydroxybenzenes is reproduced. All of the force fields used are detailed in the example LAMMPS^36,37^ input files provided in the ESI.†

**Table 1:** Binding energy 
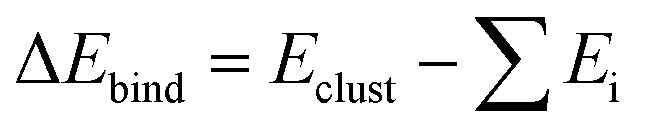
 and Gibbs free energy of binding 
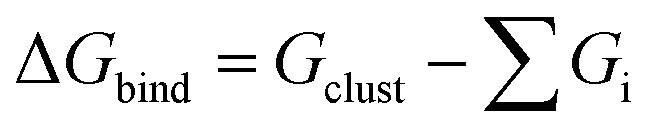
 in kcal mol^−1^ for different clusters. Δ*E*_bind,DFT_: computed by DFT using the ωB97X-D4 method with the aug-cc-pVTZ basis set (basis set superposition error (BSSE) corrected results in square brackets). Δ*E*_bind,DLPNO_: DLPNO/CCSD(T) single point energies computed at the DFT optimized geometries. Δ*E*_bind,empirical_: computed by MD simulations at *T* = 5 K using empirical force fields in LAMMPS. For comparison some literature values are also provided. Unless otherwise noted Δ*G* is calculated at *T* = 300 K and *P* = 1 atm

System	Δ*E*_bind,empirical_	Δ*E*_bind,DFT_	Δ*E*_bind,DLPNO_	Δ*G*_bind,DFT_	Literature: Δ*H*_bind_	Δ*G*_bind_
Catechol·NO_3_^−^	−27.3	−28.4[−28.1]	−29.5	−16.6(11.4^a^)	−26.2^c^	−14.1^c^
Resorcinol·NO_3_^−^	−24.9	−25.6[−25.3]	−27.0	NA	−23.1^c^	NA
Hydroquinone·NO_3_^−^	−20.3	−22.7[−22.5]	−23.6	NA	−21.7^c^	NA
4-Nitrophenol·NO_3_^−^	−29.3	−31.3[−31.0]	−31.5	NA	−31.1^d^	−22.1^d^
HNO_3_·NO_3_^−^	−29.4	−29.8[−29.5]	−29.2	−21.3(28.8^b^)	−29.7^e^	−21.5^e^

a *T* = 1000 K.

b *T* = 1600 K.

c Ref. 13.

d Ref. 11.

e Ref. 8.

### 2.1 Thermal decomposition

The optimized configurations of the nitrate ion complexes were first equilibrated for at least 200 ps at a desired target temperature using a Langevin thermostat. To ensure the complex did not dissociate during equilibration, a restraining potential was used to maintain an equilibrium distance between the centers of mass of the two molecules. All potential energy terms were cut off for interatomic distances >3.0 nm, large enough so that no intermolecular interactions were neglected while the complexes were bound.

After the equilibration phase the thermostats were turned off and the trajectory was continued in the microcanonical (constant total energy) NVE ensemble using velocity Verlet integration. The simulation time step was either 0.2 fs, or 0.5 fs in some cases where the required simulation times were exceedingly long. Testing showed no detectable difference between simulations using the different time steps.

Over the course of the simulation the distance between the centers of mass of the molecules was recorded. Post-processing was used to detect the breakup time of the complex. Each trajectory was run until such time as the centers of mass of the molecules were separated by 3.0 nm. This ensured that the cluster breakdown was irreversible. However, the actual cluster break up time was further refined by searching each trajectory backwards from this point until the molecules were separated by only 1.0 nm.

Thermal decomposition was also studied in systems with a given amount of nitrogen gas added. Gas densities were defined according to the amount of gas required for an ideal gas to have a given pressure at a temperature of 300 K. Some simulations were done at a higher temperature and therefore an increased pressure. The densities and corresponding ideal gas pressures used are given in Table 2. All simulations were run with fixed simulation box volumes. At smaller gas densities, larger simulation boxes were used to ensure a lack of finite size effects.

**Table 2:** Density of nitrogen gas introduced for a given gas pressure at *T* = 300 K, 1000 K and 1600 K for all simulation conditions in this study

Density/molec nm^−3^	*P*: 300 K/atm	1000 K	1600 K
0.0489	2	6.67	
0.02445	1	3.33	5.33
0.00611	0.25	0.83	1.33
0.00245	0.1	0.33	0.53
0.00122	0.05	0.17	0.27
0.000611	0.025	0.083	0.133
0.000245	0.01		

### 2.2 Field-driven decomposition

Field-driven decomposition was studied by preparing initial configurations according to the same procedure described above for purely thermal decomposition in nitrogen bath gas, at an initial temperature of 300 K. After equilibration, a constant electric field was applied which accelerated the negatively charged cluster in the opposite direction of the field. Because the field-induced collisions gradually add energy to the entire system, including the gas, a Langevin thermostat was applied to maintain the gas temperature at an average value of 300 K. The cluster molecules, as well as gas molecules within a radius of 5.0 nm from the center of mass of the cluster, were not included in the thermostat. This ensured that the collision dynamics of interest were not influenced by the thermostat on the gas atoms, and that the cluster temperature would only be affected by gas collisions and not by the thermostat.

## 3 Results

### 3.1 Thermal decomposition

**Fig. 2:**
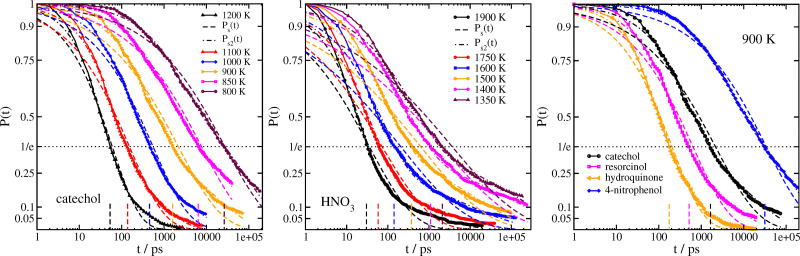
Survival probability *P*(*t*) for catechol·NO_3_^−^ and HNO_3_·NO_3_^−^ clusters at different initial temperatures, and comparison of data at *T* = 900 K for all systems except HNO_3_·NO_3_^−^. Raw data in symbols/solid lines, 2-parameter fits to eqn (1) in dashed lines, 5-parameter fits to eqn (2) in dash-dotted lines. The short vertical dashed lines indicate the time at which *P*(*t*) = 1/*e* for each system.

In Fig. 2 we show calculations of *P*(*t*), the survival probability of the cluster as a function of time. By running around 1000 trajectories for each cluster composition and initial temperature, we were able to obtain well-converged determinations of *P*(*t*).

We considered several ways of modelling *P*(*t*). The distribution was clearly not fit by a simple exponential, nor did it show clear separation of timescales indicative of a bi-exponential distribution. Instead, the best fitting function we were able to find was to use a stretched exponential distribution,^38^ also known as a Kohlrausch–Williams–Watts (KWW) distribution,^39,40^ which has sometimes been used to describe similar survival time distributions,^41^ albeit usually in different contexts, *eg.* dielectric relaxation in solids. We use either a single stretched exponential model,

1 *P*_s_(*t*) = e^−(*t*/*τ*)*β*^,

or a sum of two stretched exponentials,2
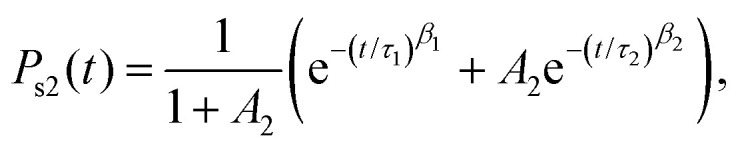
with the added parameter *A*_2_ introduced to determine the relative contributions of the two stretched exponentials, along with ensuring the correct initial condition 
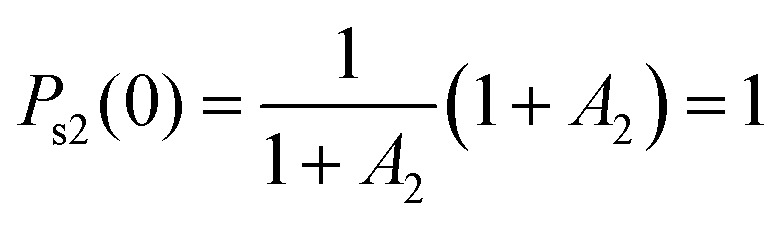
. Fits to eqn (1) and (2) are shown along with the raw data for catechol·NO_3_^−^ and HNO_3_·NO_3_^−^, as well as a comparison of the four functionalized benzene systems at *T* = 900 K, in Fig. 2, while the fit parameters are shown for some systems in Table 3 (tables of the rest of the fit parameters, as well as figures of the rest of the fits and the raw data, can be found in the ESI†).

**Table 3:** Fit parameters for purely thermal decomposition of catechol·NO_3_^−^ and HNO_3_·NO_3_ clusters. *Fits for HNO_3_·NO_3_^−^ at *T* = 1350 K are greatly affected by the missing long tail at longer times

System	*T*/K	Eqn (1): *τ*/ps	*β*	Eqn (2): *A*_2_	*τ*_1_/ps	*β*_1_	*τ*_2_/ps	*β*_2_	〈*τ*_s2_〉/ps
Catechol·NO_3_^−^	1200	59.7	0.661	0.384	36.1	0.928	262.1	0.687	121
	1100	151.7	0.515	0.720	64.8	0.881	579.3	0.555	447
	1000	548.2	0.503	0.442	229.2	0.707	3881	0.584	2055
	900	2165	0.421	0.722	506.3	0.696	14925	0.477	1.408 × 10^4^
	850	7651	0.424	0.978	1034	0.664	43697	0.580	3.474 × 10^4^
	800	27319	0.411	1.717	2209	0.704	88393	0.513	1.075 × 10^5^
HNO_3_·NO_3_^−^	1900	35.5	0.401	0.296	23.8	0.751	644.8	0.353	741
	1750	72.7	0.363	0.304	41.6	0.648	1953	0.336	2695
	1600	207.1	0.270	0.516	67.8	0.615	5693	0.283	2.368 × 10^4^
	1500	629.0	0.267	0.804	107.0	0.655	8746	0.309	3.179 × 10^4^
	1400	2062	0.252	0.569	274.0	0.544	102072	0.302	3.331 × 10^5^
	1350 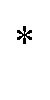	3668	0.256	0.623	365.9	0.516	147880	0.338	3.238 × 10^5^

A value of the exponent *β* = 1 would indicate simple exponential behaviour. It is clear that *β* deviates significantly from 1 for all systems. For a simple exponential, the mean relaxation time 〈*τ*〉 would be simply equal to the relaxation timescale *τ*, since 
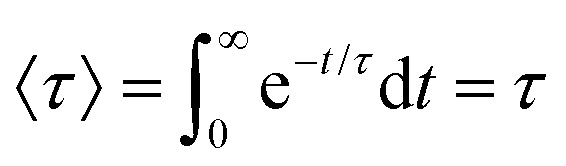
. A rough idea of the best value of *τ* for a single simple exponential fit would be the value of *t* at which *P*(*t*) = 1/*e*, and for comparison this value for each system is also shown in Fig. 2.

For stretched exponentials however, the mean relaxation time has a more complex form:3
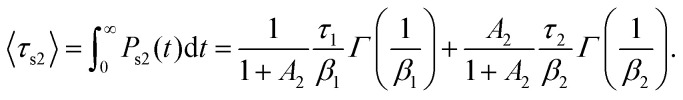


The stretched exponential distribution has a very long tail, which leads to greatly increased values of the mean relaxation times compared to what would be obtained with a single exponential distribution.^38,42^

**Fig. 3:**
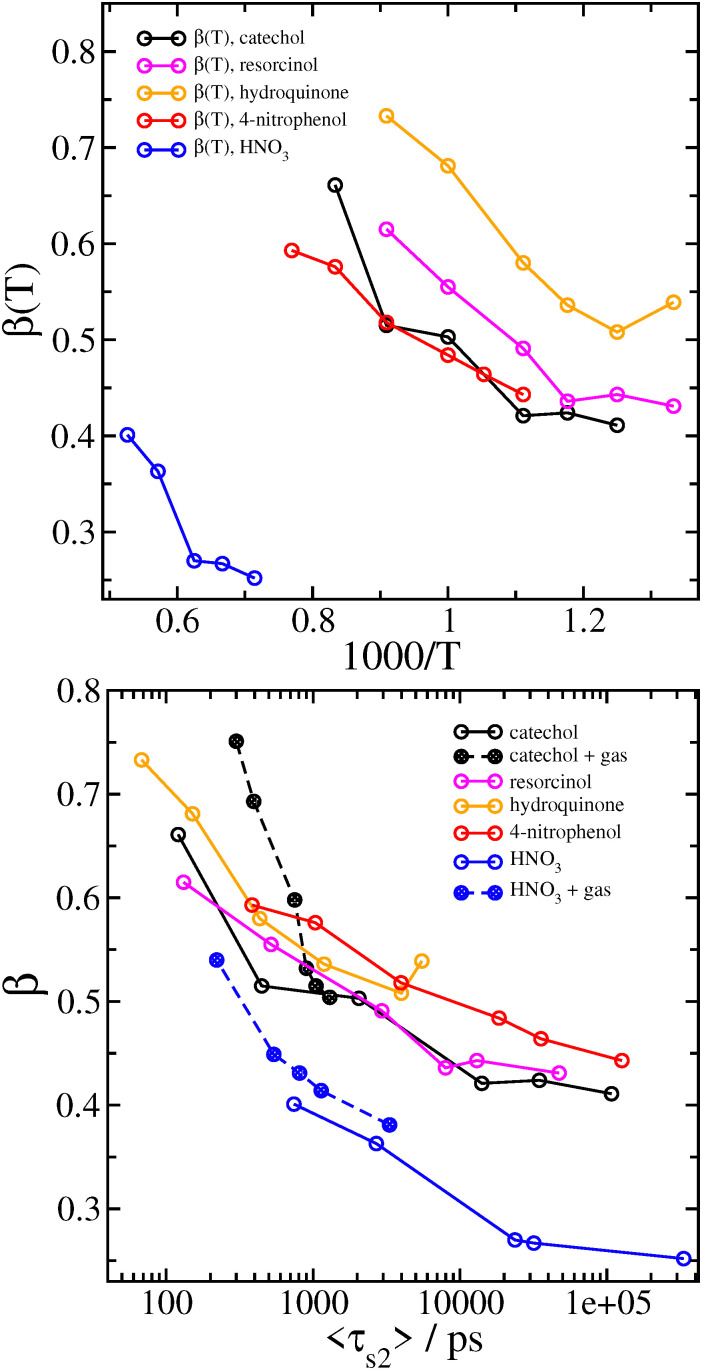
Variation in *β* in fits of *P*(*t*) to eqn (1) with system temperature (top), and with 〈*τ*_s2_〉 computed from eqn (3), also including simulations with a N_2_ bath gas (bottom).

In terms of understanding the trends among different systems and conditions, the single stretched exponential fits (eqn (1)) are most useful. Other than the obvious increase in the timescale parameter *τ* as temperature is decreased, we also see that the stretching exponent *β* lowers as temperature is lowered. We can rationalize this behaviour as a consequence of the fact that temperature is not constant in a small molecular system. Although on average the temperature of the cluster is close to the targeted equilibrated temperature, the average temperature of individual trajectories varies greatly. We note also that the HNO_3_·NO_3_^−^ cluster has very low values of *β*; since it has less degrees of freedom, its average temperature varies more. The variation in *β* with initial temperature is shown in Fig. 3.

In terms of accurately computing the mean relaxation time 〈*τ*_s2_〉, it is important to fit the raw data with as little error as possible. The best estimates come from fitting the simulated results for *P*(*t*) using two stretched exponentials (eqn (2)) and then integrating with eqn (3).

We mainly introduce the fitting function eqn (2) to allow more accurate computation of 〈*τ*_s2_〉, and so we are not too concerned with analysing the values of the individual fitting parameters. We can see that in general, *τ*_1_ < *τ*_2_ and *β*_1_ > *β*_2_, so that the distribution of multiple stretched exponentials is just an extension of bi-exponential distributions, with two different timescales. We also note that the single value of *β* used to fit eqn (1) is always smaller than both of the values of *β*_1_ and *β*_2_ used to fit eqn 2. Similarily, stretched exponential distributions have been shown to arise from several different simple exponential distributions (*i.e.* with *β* = 1) combining to produce a broader distibution with *β* < 1.^42^

Values of 〈*τ*_s2_〉 derived from eqn (3) are included in Table 3. When the variation in *β* (from the single stretched exponential fit) with 〈*τ*_s2_〉 for each system and initial temperature is plotted (Fig. 3), we can see that for the larger complexes with the functionalized benzene analyte molecules, which have nearly the same number of degrees of freedom, all of the data collapses on a similar curve.

Since we have 〈*τ*_s2_〉 as a function of temperature, an obvious next step is to check if the results obey an Arrhenius-type relation by fitting the values of 〈*τ*_s2_〉 to an expression of the form

4 〈*τ*_s2_〉 (*T*) = *A* exp(*B*/*k*_B_*T*).

**Fig. 4:**
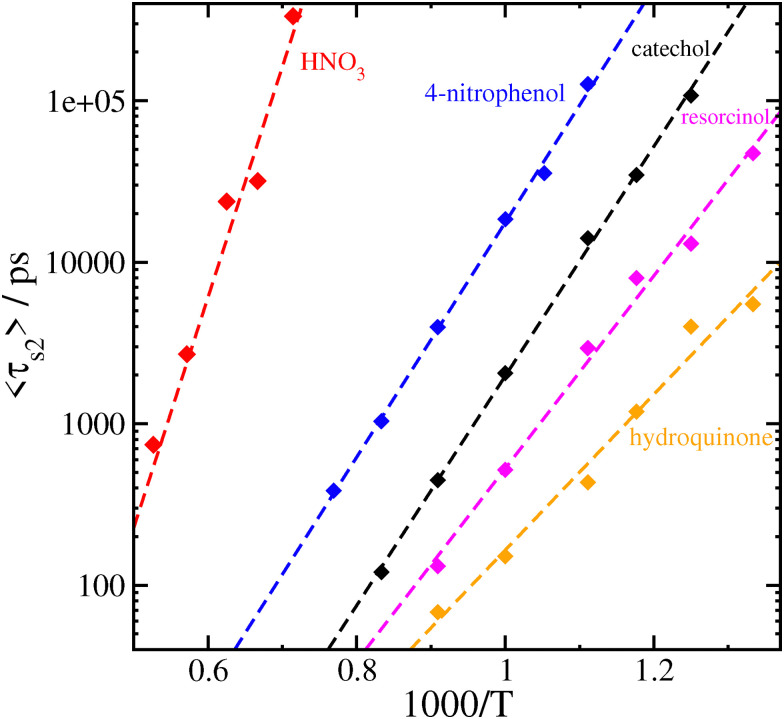
Fit of the mean relaxation time 〈*τ*_s2_〉 (*T*) to an Arrhenius relation (eqn (4)). The fit for HNO_3_·NO_3_^−^ does not include the data at *T* = 1350 K.

We show the results of fitting our data to eqn (4) in Fig. 4. In all cases, it appears that the Arrhenius relation empirically fits the temperature dependence well, with fitted parameters *A* and *B* listed for each system in Table 4. The exact meaning of the fitted parameters is unclear; while it may be tempting to interpret *B* as some sort of “activation energy” as in the original formulation of Arrhenius, and the numerical values are reasonable, for the purposes of this study we will avoid speculation. Purely as an empirical relation, eqn (4) allows us to extrapolate our MD data, collected at elevated temperatures, to more atmospherically or experimentally relevant temperatures. Table 4 also shows the extrapolated values for the mean relaxation times of our systems obtained for *T* = 300 K.

**Table 4:** Fitted parameters *A* and *B* in the Arrhenius relation (eqn (4)) for each system studied, and extrapolated mean relaxation times at *T* = 300 K for thermal decomposition in vacuum

System	*A*/fs	*B*/kcal mol^−1^	〈*τ*_s2_〉 (*T* = 300 K)
Catechol	0.155	32.51	870 days
Resorcinol	0.574	27.29	12.1 hours
Hydroquinone	2.50	22.09	29 s
4-Nitrophenol	9.61	33.23	500 years
HNO_3_	0.0167	65.28	2 × 10^23^

We can compare the inverse of the extrapolated survival time with an estimate of the dissociation rate *γ* derived from detailed balance.^43–45^ Using values of the association rate *β* = 10^−9^ cm^3^ s^−1^ and binding free energies listed in Table 1 computed at a reference pressure of 1 atm and temperature of 300 K, we obtain *γ* = 0.02 s^−1^ for catechol–nitrate dissociation and *γ* = 7.43 × 10^−6^ s^−1^ for HNO_3_–nitrate. These are far in excess of estimates based on the extrapolated *T* = 300 K survival times derived in this work (*γ* = 1.33 × 10^−8^ s^−1^ and 1.65 × 10^−31^ s^−1^ for catechol and nitric acid, respectively). Our extrapolated survival times are very long for some of the clusters (the nitric acid–nitrate cluster being far in excess of the age of the universe) and should be taken with a grain of salt; however, we note that these would be significantly reduced by surrounding gas, as discussed below.

Instead of relying on the extrapolation of our MD results to lower temperatures, we may instead attempt to recalculate the dissociation rate at high temperatures using quantum chemical methods. The relevant values of Δ*G*_bind_ are included in Table 1. Using the same value of the association rate *β* = 10^−9^ cm^3^ s^−1^, we now obtain *γ* = 2.28 × 10^12^ s^−1^ for catechol–nitrate clusters at 1000 K, and *γ* = 3.94 × 10^13^ s^−1^ for HNO_3_–nitrate at 1600 K. For comparison, the estimates we have in this work based on the inverse of 〈*τ*_s2_〉 are *γ* = 4.87 × 10^8^ s^−1^ for catechol–nitrate at 1000 K and *γ* = 4.22 × 10^7^ s^−1^ for HNO_3_–nitrate at 1600 K. Similar to the 300 K results, dissociation rates from detailed balance and quantum chemical calculations are orders of magnitude higher than those we estimate in this work from molecular dynamics. Such large discrepancies between different theoretical approaches are not uncommon when estimating rate constants, but are still worth examining further. We discuss some possible reasons for the discrepancies and suggest ways to resolve them in our Conclusions.

**Fig. 5:**
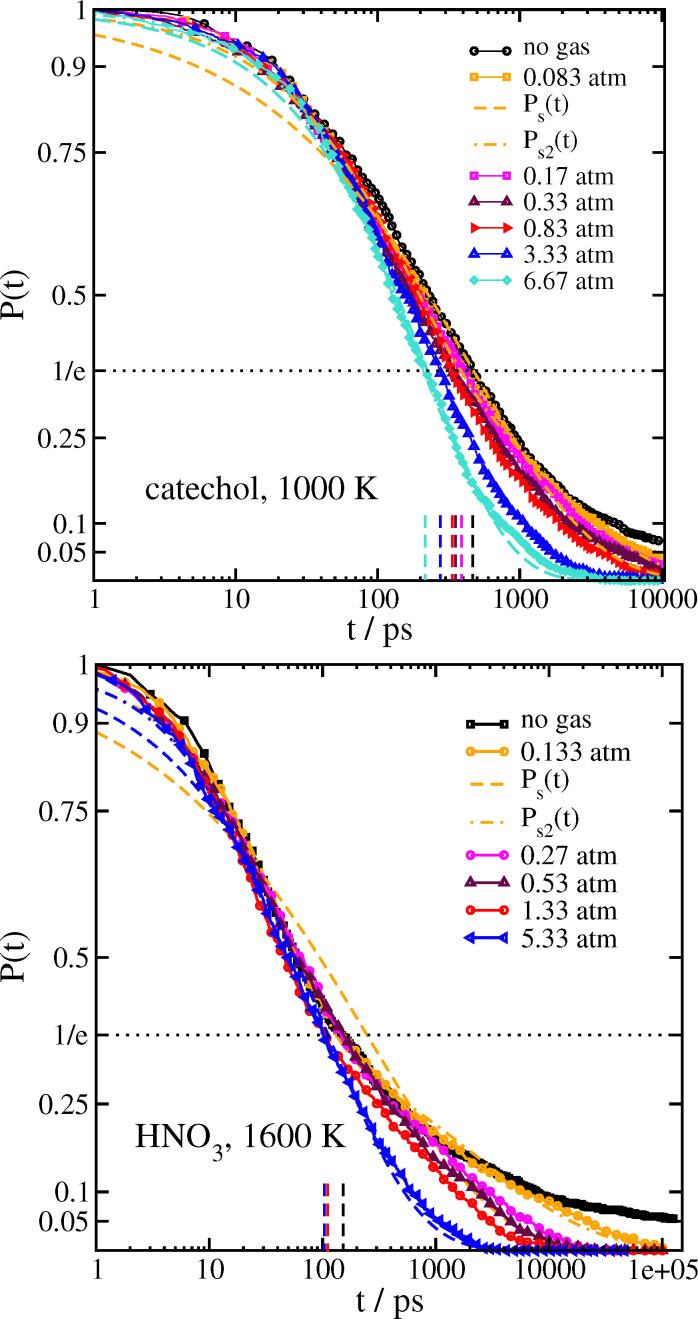
Survival probability with surrounding gas. (top) catechol·NO_3_ clusters at 1000 K. (bottom) HNO_3_·NO_3_^−^ clusters at 1600 K. Fits to eqn (1) and (2) are only shown for the lowest and highest gas densities. Short vertical dashed lines indicate the time at which *P*(*t*) = 1/*e* for each system.

**Fig. 6:**
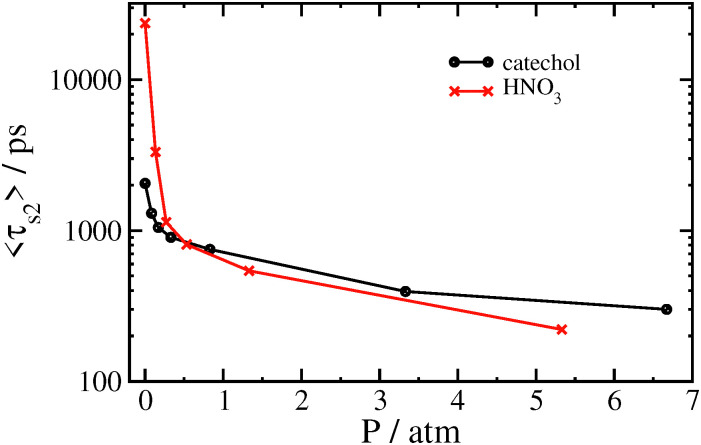
Variation in 〈*τ*_s2_〉 with gas pressure for thermal decomposition of catechol–nitrate and HNO_3_–nitrate clusters.

When a surrounding gas is added to the system, collisions with gas molecules lead to more effective thermalization. This has the main effect of increasing the value of the stretching exponent closer to *β* = 1 corresponding to a simple exponential, as shown in Fig. 5 and Table 5 for catechol- and HNO_3_–nitrate clusters. It follows that the mean survival times of the clusters are significantly reduced by the presence of gas. In practice, collisions with gas molecules warm up the initially colder clusters which are contributing to the very long tail in the survival time distribution. The effect of the gas on 〈*τ*_s2_〉 is shown graphically in Fig. 6. The variation in *β* with 〈*τ*_s2_〉 for the simulations with gas is included in Fig. 3. The increase in *β* and decrease in 〈*τ*_s2_〉 are especially dramatic in the HNO_3_ case, as the gas collisions play a larger role in thermalizing the smaller molecule. Even at the lowest gas density, the mean relaxation time 〈*τ*_s2_〉 computed from eqn (3) is reduced by an order of magnitude, and therefore the dissociation rate should be increased by a similar factor. It may also be worth noting that the shape of *P*(*t*) in the bath gas does not change much for low values of *t*; the main influence of the gas is in causing very long-lived clusters to decompose sooner. In principle, we might expect the gas to also be cooling down initially hot clusters; however, these hot clusters decompose so quickly that collisions with gas do not have time to thermalize the cluster to any great extent before they decompose.

**Table 5:** Fit parameters for purely thermal decomposition of catechol·NO_3_^−^ and HNO_3_·NO_3_^−^ clusters in the presence of nitrogen gas at different pressures

System	P/atm	Eqn (1): *τ*/ps	*β*	Eqn 2: *A*_2_	*τ*_1_	*β*_1_	*τ*_2_	*β*_2_	〈*τ*_s2_〉/ps
Catechol·NO_3_^−^ (*T* = 1000 K)	0.083	472.0	0.504	0.873	142.9	0.794	1705	0.593	1301
	0.17	440.9	0.515	1.084	126.0	0.791	1270	0.607	1048
	0.33	382.0	0.532	1.116	122.4	0.845	1028	0.588	901
	0.83	377.4	0.598	0.643	170.1	0.795	1226	0.672	750
	3.33	280.2	0.693	1.111	119.3	0.893	559.4	0.795	395
	6.67	223.3	0.751	0.664	126.0	0.916	502.5	0.831	300
HNO_3_·NO_3_^−^ (*T* = 1600 K)	0.133	246.4	0.381	0.828	44.2	0.841	2096	0.394	3318
	0.27	207.2	0.414	0.705	43.0	0.719	1493	0.533	1136
	0.53	189.3	0.431	1.107	34.5	0.804	765.4	0.504	810
	1.33	143.7	0.449	1.002	27.0	0.933	630.5	0.557	541
	5.33	111.9	0.540	1.732	23.5	0.863	240.0	0.640	221

It is worth emphasizing that multiple interactions between gas molecules and the nitrate complex can be expected to occur before these collisions can thermalize the complex. The situation is somewhat different in the field-driven case we discuss next, where the complex may remain well out of thermal equilibrium, especially in high field and low pressure conditions.

### 3.2 Field-driven decomposition

In the ionization inlet of CIMS instruments, conditions are meant to approximately match atmospheric conditions. This means that the pressure is on the order of 1 atm, with comparatively low fields used to move the ionized analytes into the rest of the apparatus. Meanwhile, much larger fields (∼0.1 V μm^−1^) are applied in the mass spectrometry drift tube, but in this part of the apparatus the pressure is typically very low (∼10^−3^ atm).

Due to limitations on the simulation time and the system size, our simulations of field-driven decomposition have been limited to a minimum field strength of 0.05 V μm^−1^ and a minimum pressure of 0.01 atm. The field strength is much larger than in the ionization inlet, and the pressure is much higher than in the mass spectrometer itself. This makes exact comparisons between our simulations results and the real experiments somewhat awkward, however we can still draw some relevant conclusions.

For the field-driven decomposition we focused our attention on the catechol–nitrate cluster, looking at a wide range of different gas densities. For comparison we also studied the hydroquinone- and HNO_3_–nitrate clusters at two different gas densities. We ran multiple trajectories to obtain well-converged averages. In most cases, we ran 100 trajectories for each combination of field strength and gas density. In some cases, the simulation time required to consistently observe cluster decomposition exceeded 1 μs, and so we only ran 25 trajectories.

**Fig. 7:**
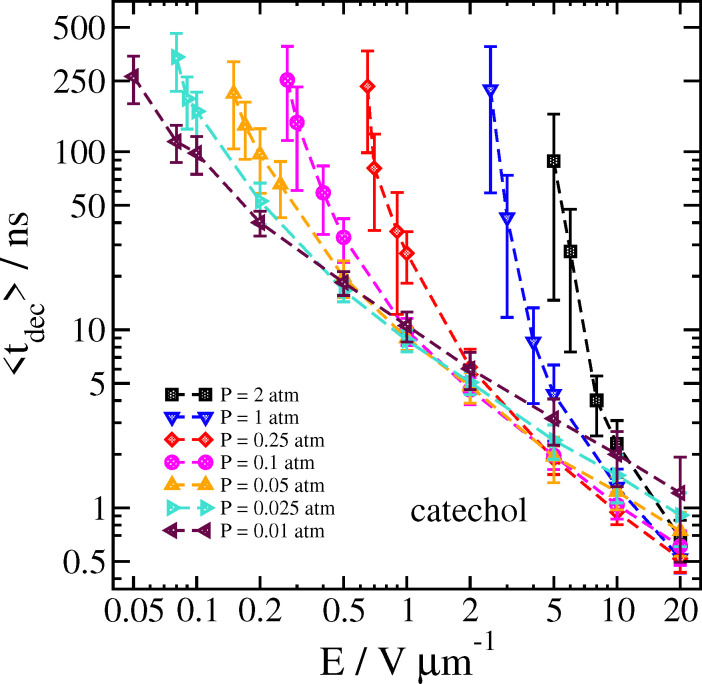
Time 〈*t*_dec_〉 at which the catechol–nitrate cluster decomposes as a function of field strength for different pressures at *T*_gas_ = 300 K. Error bars are one standard deviation.

When the cluster is accelerated by the electric field through a gas, collisions with the gas eventually lead to cluster decomposition. In Fig. 7 we show how the average cluster decomposition time 〈*t*_dec_〉 for the catechol–nitrate cluster depends on both the gas density and the applied field strength. At low density and high field, there is a rough power-law relationship between the field strength and 〈*t*_dec_〉, as indicated by the linear appearance of the log–log plot. As the field strength is lowered, however, we see that the decomposition time increases greatly, eventually rising higher than the maximum simulation time we can manage.

**Fig. 8:**
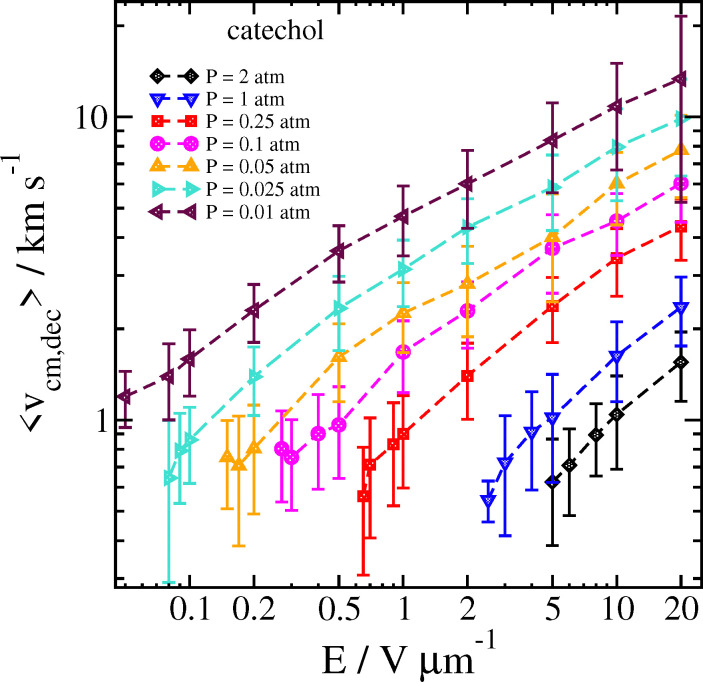
Velocity of the catechol–nitrate cluster 2 ps before decomposition is detected as a function of field strength for different pressures at *T*_gas_ = 300 K. Error bars are one standard deviation.

**Fig. 9:**
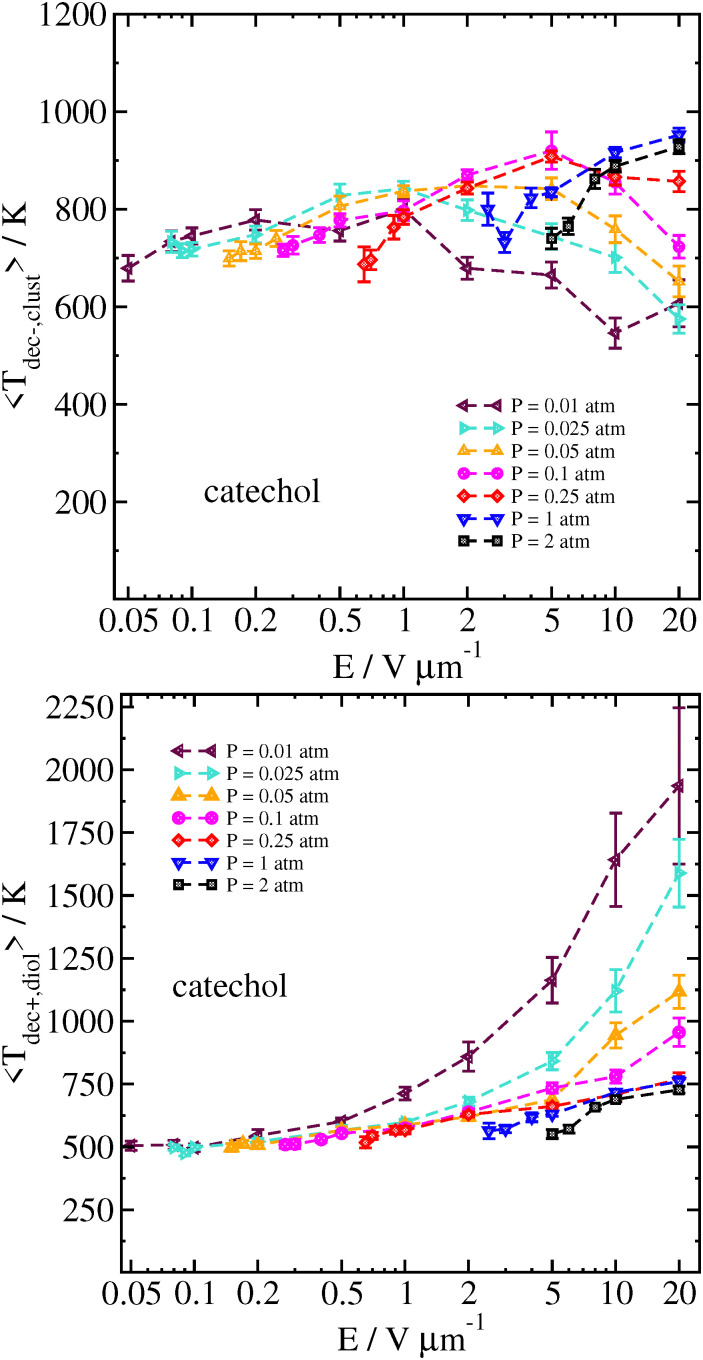
Temperature of the catechol–nitrate cluster just before decomposition (top), and catechol at the time decomposition is detected (bottom), as a function of field strength for different pressures at *T*_gas_ = 300 K. Error bars are the standard error of the mean temperatures over all trajectories.

The reason for this sharp increase can be better understood by also examining the velocity of the cluster 2 ps before the decomposition is detected, as well as the temperature of the cluster and the separated analyte molecule just before and after the decomposition, respectively. These results can be seen in Fig. 8 and 9.

At high field strength and low gas density, the average cluster temperature just before decomposition is reduced, and the temperature of the analyte immediately after decomposition is significantly increased. This shows that the cluster is not fully thermalized by the gas. In sharp contrast with the results for purely thermal decomposition, where the gas mainly serves to thermalize the cluster, a single very energetic collision can be sufficient to induce decomposition in a cluster which is still rather cold.

A similar effect has been shown for simulations of solvent loss during electrospray ionization; the effect was more important in that study due to the relatively lower binding energy of water or other solvent to the ion (*ca.* 5–7 kcal mol^−1^).^21,46^ In Ref. 21 it was shown that one could even assume that, at low gas density and high field, the first collision between the cluster and gas was usually sufficient to cause solvent loss, and therefore basic physics could predict the cluster velocity at breakup from knowing the field strength, the cluster mass and the time of initial solvent loss. In the current study, the larger binding energy of nitrate complexes makes such a simple model inadequate; at least a few collisions are needed to cause the complex to dissociate. On the other hand, in the near-vacuum conditions in the mass spectrometer drift tube, our data shows that we can expect that even very strongly bound clusters are significantly non-thermalized.

In our simulations at higher gas densities, the systems seem to be well-thermalized at the time of decomposition. Here, at high field the decomposition temperature of the cluster is highest at the highest field strength. It may take some time for random fluctuations to cause the cluster to dissociate; if the field strength is higher, then the higher velocity and more energetic collisions will tend to make the cluster hotter on average. We also note that the analyte molecule's temperature is significantly colder after the cluster decomposition at lower field strength and/or higher pressure, owing to the energy loss associated with breaking the hydrogen bond(s) with the nitrate ion.

At the smallest field strengths we have simulated, cluster decomposition is only observed in gas of lower density. This again indicates that at field strengths typically used in the ionization inlet of CIMS instruments (∼10 V cm^−1^),^18^ cluster decomposition is unlikely to be an important factor. However, in the so-called cluster fragmentation regime inside the MS instrument where higher field strengths are applied, our results may serve as a useful guide for calibration experiments.^22,23,47,48^ In particular, it may be interesting to use gas of different density in order to more carefully tune the cluster decomposition dynamics.

**Fig. 10:**
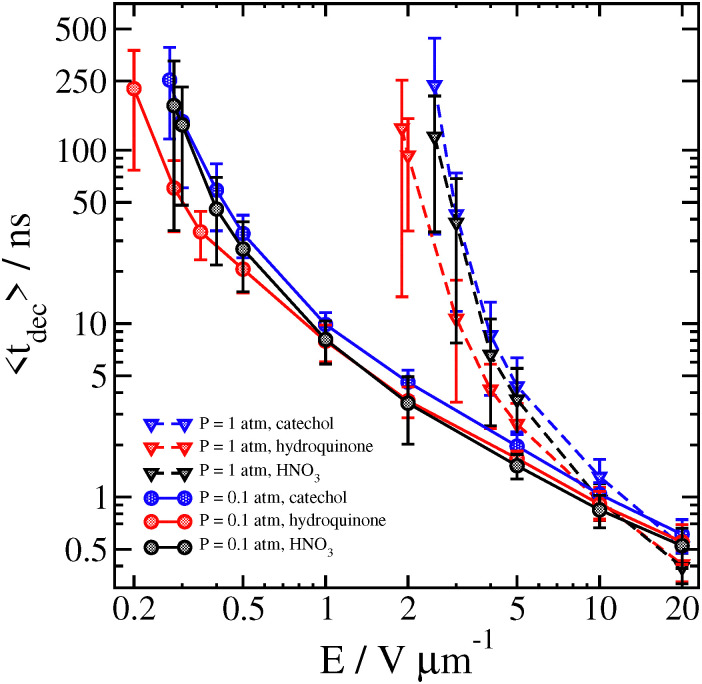
Average time 〈*t*_dec_〉 at which different clusters decompose as a function of field strength for *P* = 0.1 atm and *P* = 1 atm at *T*_gas_ = 300 K. Error bars are one standard deviation.

**Fig. 11:**
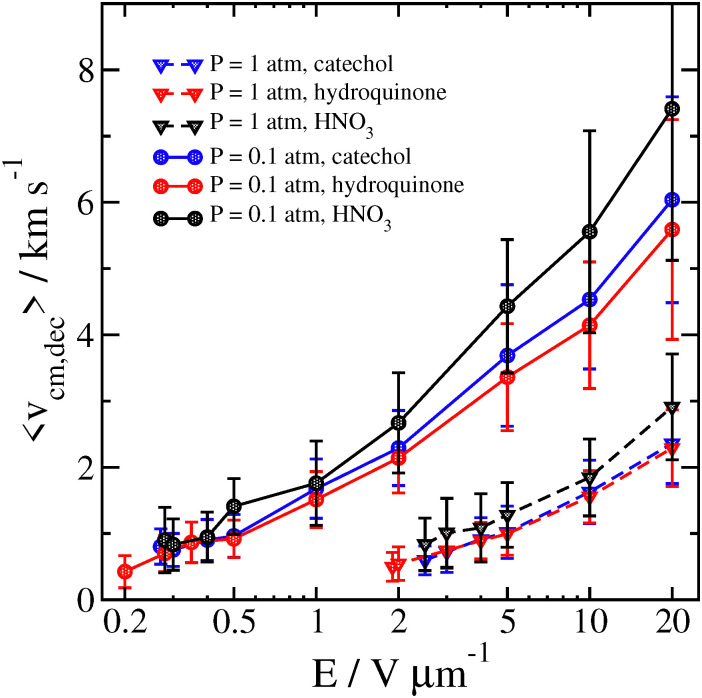
Average velocity of different clusters 2 ps before decomposition is detected as a function of field strength for *P* = 0.1 atm and *P* = 1 atm at *T*_gas_ = 300 K. Error bars are one standard deviation.

**Fig. 12:**
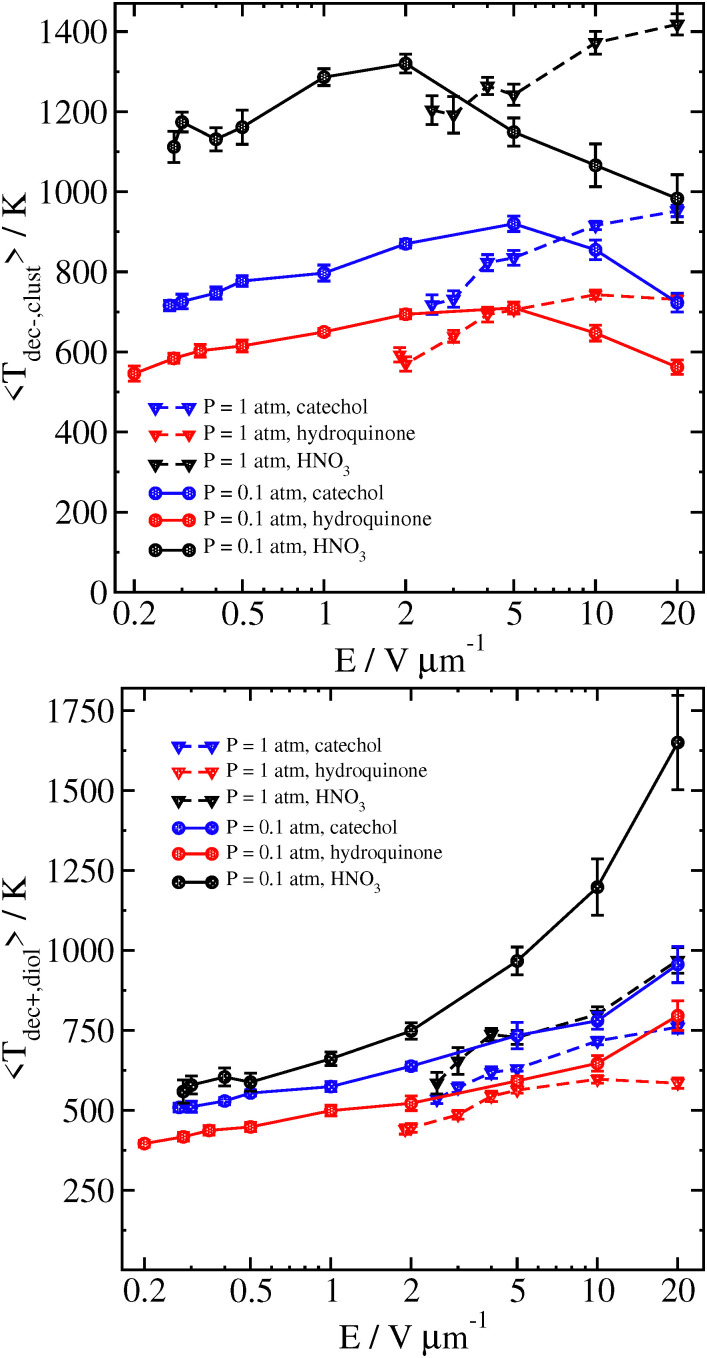
Temperature of different clusters just before decomposition (top), and each analyte at the time decomposition is detected (bottom), as a function of field strength for *P* = 0.1 atm and *P* = 1 atm at *T*_gas_ = 300 K. Error bars are the standard error of the mean temperatures over all trajectories.

We compare some results between different clusters for field-driven decomposition in Fig. 10, 11 and 12. Qualitatively, the different clusters behave similarly to the catechol case. The hydroquinone case tends to fall apart faster and at lower velocity compared to the catechol owing to the reduced binding energy. The nitric acid cluster, on the other hand, although it has comparable binding energy to catechol, is considerably lighter. This leads to faster decomposition compared with the catechol cluster, especially at lower pressures and field magnitude since it will be accelerated comparatively faster and undergo more energetic collisions at the same field strength.

## 4 Conclusions

We have presented the results of a large number of simulations of ion–molecule breakup, including purely thermal decomposition, as well as field-induced decomposition due to collisions with gas. Our approach highlights the importance of considering these systems as an ensemble of gas-phase clusters, with a range of different thermal energies. This is similar to previous work where stretched exponentials have been shown to describe a distribution of different decay processes, each one of which is a simple exponential.^42^

The very long-tailed distribution of cluster survival lifetimes described in this work by stretched exponentials is fundamentally different than what would be predicted from a treatment which assumes each cluster has a well-defined exact temperature. Such fundamental issues as how to treat the thermal properties of an isolated gas-phase system correctly in molecular dynamics simulations continue to be important research topics even today.^49–51^

Comparing the dissociation rate *γ* predicted from our simulations, we find that our estimates of *γ* are orders of magnitude lower than those derived from the more standard detailed balance approach. Other approaches have also noted that the detailed balance estimates may be dramatically underestimating the true value of *γ*, albeit not by such a large factor.^52^ Although our results fit an Arrhenius relation well, suggesting that extrapolation to lower temperatures should be possible, it is likely that extrapolation all the way down to 300 K (where we expect the detailed balance estimate to be accurate) is unreliable. On the other hand, using the detailed balance approach may be unreliable at higher temperatures,^53^ and besides the quasi-RRHO calculation of vibrational frequencies used as a default in ORCA is also not reliable at high temperature, requiring advanced methods such as vibrational perturbation theory which can account for vibrational anharmonicity.^54^ Nevertheless, further investigation of this discrepancy is required, perhaps by using the MD methods applied in this work to study less strongly bound clusters where results for binding times could be obtained at closer to ambient temperatures. Simulations of less strongly bound clusters might also be easier to model with simple physics, as was used previously to good effect in simulations of solvent loss in ESI-MS.^21^

Our results for field-driven cluster breakup demonstrate that, at the lower field strengths typically present in the IMR region for CIMS studies of trace gases, the cluster decomposition is unlikely to be an important factor. However, there is interest in the experimental atmospheric chemistry community in using higher electric fields in order to explore the fragmentation of clusters. Mass spectrometry relies on accurate calibration, however the extent of ion fragmentation is generally not well known, and difficult to quantify. Our new simulation results can form the basis for better constraining mass spectrometry experiments, and guiding the development of new experimental methods.

Our observation that the total mass of the cluster, as well as the associated higher number of degrees of freedom, can influence the decomposition is also noteworthy. The main application area of NO_3_^−^ CIMS is in quantifying the concentration of highly functionalized molecules that act as direct secondary aerosol precursors in ambient air. According to the current results, the heat sink afforded by the large molecular structures of these molecules is a significant feature leading to very stable ion–molecule adducts and consequently our results contribute to explaining the extreme sensitivity characteristic of NO_3_^−^ ionization.

## Data availability

LAMMPS input files to regenerate the trajectories are provided in the ESI.† We have also provided the Grace .agr files containing the computed survival times *P*(*t*) and the various fits to eqn (1) and (2) in the ESI.† Due to the size of the actual MD trajectories, we have not made them available, but they can be provided as requested.

## Conflicts of interest

There are no conflicts to declare.

## Supplementary Material

CP-027-D5CP00908A-s001

CP-027-D5CP00908A-s002

CP-027-D5CP00908A-s003
