# Supplementary material for: Molecular dynamics simulations of atmospherically relevant molecular clusters: a case study of nitrate ion complexes
Source: Phys Chem Chem Phys. 2025 May 1;27(20):10804–14. doi: 10.1039/d5cp00908a (PMC12067874; doi:10.1039/d5cp00908a)
Supplement: CP-027-D5CP00908A-s001 [file CP-027-D5CP00908A-s001.pdf]

Supplementary Information (SI) for Physical Chemistry Chemical Physics.
This journal is © the Owner Societies 2025

Supporting Information: Molecular dynamics simulations of atmospherically relevant molecular clusters: A case study of nitrate ion complexes

Christopher David Daub,*,† Theo Kurtén,‡ and Matti P. Rissanen*,†,‡

†*Aerosol Physics Laboratory, Physics Unit, Faculty of Engineering and Natural Sciences, Tampere University, Tampere 33720, Finland*

‡*Department of Chemistry, University of Helsinki, P.O. Box 55, Helsinki 00014, Finland*

E-mail: christopher.daub@tuni.fi; matti.rissanen@tuni.fi

Plots of $P(t)$ and data tables for systems not shown in the main text

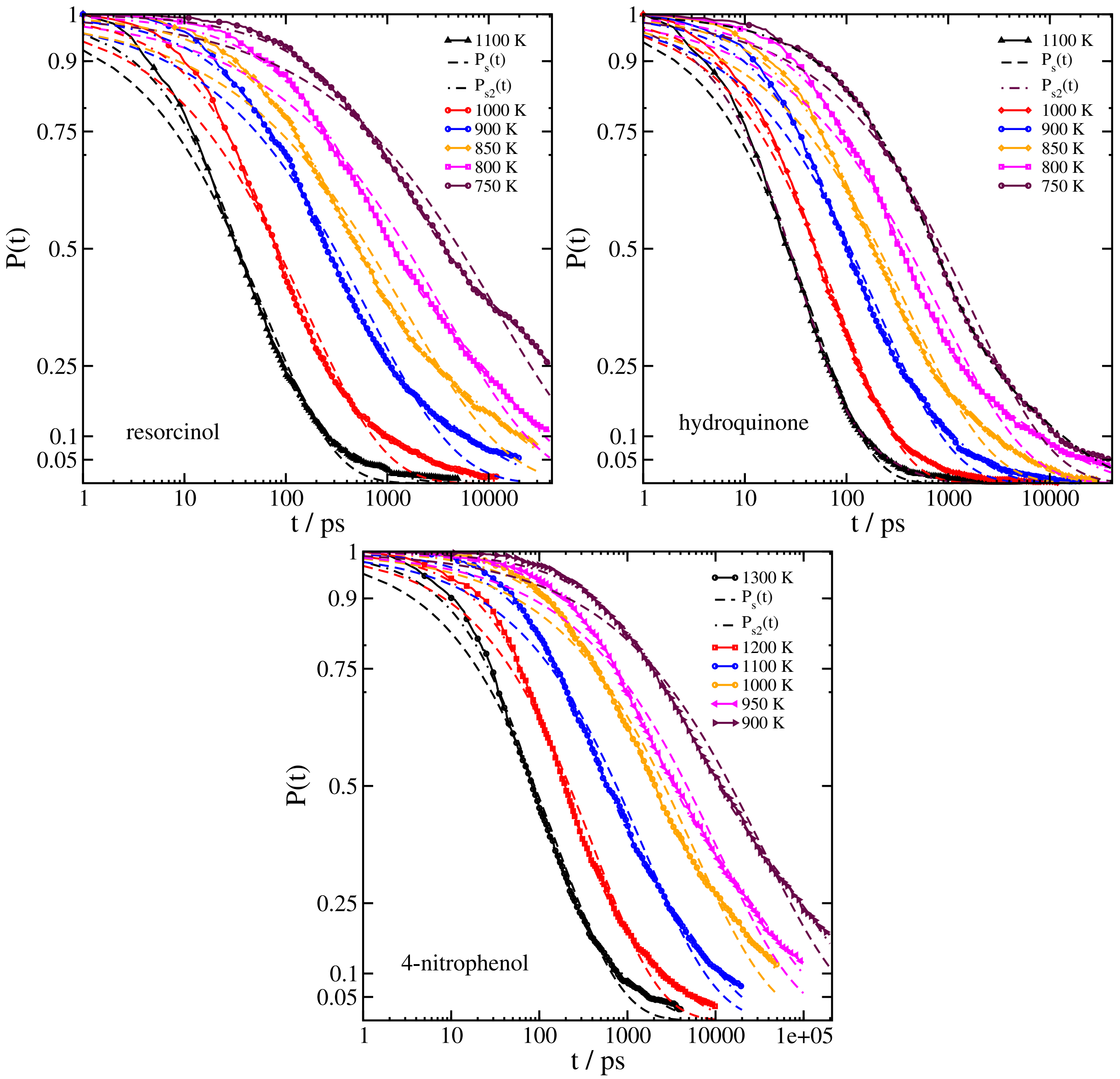

Figure S1: Plots of the survival probability $P(t)$, and fits to Equations 1 and 2 in the main text, for purely thermal decomposition of resorcinol$\cdot NO_3^-$, hydroquinone$\cdot NO_3^-$ and 4-nitrophenol$\cdot NO_3^-$ clusters. Plots for other systems are shown in the main text.

Table S1: **Fit parameters in Equations 1 and 2 in the main text for purely thermal decomposition of resorcinol·NO_3^-, hydroquinone·NO_3^- and 4-nitrophenol·NO_3^- clusters. Data for other systems are shown in the main text.**

System	T / K	Eqn.1: τ / ps	β	Eqn.2: A_2	τ_1 / ps	β_1	τ_2 / ps	β_2	$\langle\tau_{s2}\rangle$ / ps
resorcinol·NO_3^-	1100	61.1	0.615	0.371	39.5	0.838	251.1	0.612	131
	1000	159.9	0.555	0.441	83.9	0.857	897.7	0.560	518
	900	638.2	0.491	0.519	247.2	0.751	4319	0.521	2937
	850	1515	0.436	0.714	368.9	0.726	9828	0.517	7976
	800	3571	0.443	1.248	538.8	0.782	13161	0.541	1.304×10^4
	750	12105	0.431	0.979	1342	0.716	72711	0.686	4.728×10^4
hydroquinone·NO_3^-	1100	44.9	0.733	0.304	32.7	0.922	146.9	0.724	68.1
	1000	85.9	0.681	0.286	60.5	0.843	344.3	0.679	151
	900	205.3	0.580	0.941	86.1	0.949	549	0.615	434
	850	411.7	0.536	0.625	165.5	0.821	1809	0.588	1189
	800	836.7	0.508	0.495	332.0	0.768	6022	0.519	3991
	750	1721	0.539	0.691	638.7	0.780	7268	0.549	5506
4-nitrophenol·NO_3^-	1300	163.6	0.593	0.414	90.8	0.816	762.0	0.635	385
	1200	414.6	0.576	0.625	176.6	0.869	1659	0.621	1036
	1100	1524	0.518	1.089	370.5	0.847	4878	0.606	3971
	1000	5600	0.484	0.848	1171	0.745	27009	0.626	1.844×10^4
	950	10424	0.464	1.371	1485	0.817	35332	0.549	3.558×10^4
	900	34085	0.443	1.225	4087	0.699	141822	0.578	1.265×10^5

Input files for LAMMPS

The attached ZIP file contains "input" and "data" files for running LAMMPS simulations with all of the required initial configurations and force field parameters. The initial temperature can be changed as needed. By changing the random number seeds for initial velocity generation and the Langevin thermostat used for equilibration, multiple trajectories can be generated and the data post-processed to detect the cluster decomposition and perform other data analyses.

For electric field-driven decomposition in nitrogen bath gas, "input" and "data" files containing initial configurations used for simulations of the catechol-nitrate cluster with a gas density of 0.00245 molecules/nm (equivalent to $P = 0.1$ atm at $T = 300$ K) are provided. The field strength can be changed as needed. The random number seeds can once again be altered to generate multiple independent trajectories, followed by similar data analyses as in the cases with no added gas or electric field.

Grace .agr files

Grace version 5.1.25 was used to make most of the graphs. We have provided the .agr files used herein. These contain the raw data for survival times $P(t)$ generated from analysis of the raw trajectories as described in the main text, as well as all of the various fittings.
